# Simulated impacts of ankle foot orthoses on muscle demand and recruitment in typically-developing children and children with cerebral palsy and crouch gait

**DOI:** 10.1371/journal.pone.0180219

**Published:** 2017-07-13

**Authors:** Michael Rosenberg, Katherine M. Steele

**Affiliations:** Department of Mechanical Engineering, University of Washington, Seattle, Washington, United States of America; Northwestern University, UNITED STATES

## Abstract

Passive ankle foot orthoses (AFOs) are often prescribed for children with cerebral palsy (CP) to assist locomotion, but predicting how specific device designs will impact energetic demand during gait remains challenging. Powered AFOs have been shown to reduce energy costs of walking in unimpaired adults more than passive AFOs, but have not been tested in children with CP. The goal of this study was to investigate the potential impact of powered and passive AFOs on muscle demand and recruitment in children with CP and crouch gait. We simulated gait for nine children with crouch gait and three typically-developing children with powered and passive AFOs. For each AFO design, we computed reductions in muscle demand compared to unassisted gait. Powered AFOs reduced muscle demand 15–44% compared to unassisted walking, 1–14% more than passive AFOs. A slower walking speed was associated with smaller reductions in absolute muscle demand for all AFOs (r^2^ = 0.60–0.70). However, reductions in muscle demand were only moderately correlated with crouch severity (r^2^ = 0.40–0.43). The ankle plantarflexor muscles were most heavily impacted by the AFOs, with gastrocnemius recruitment decreasing 13–73% and correlating with increasing knee flexor moments (r^2^ = 0.29–0.91). These findings support the potential use of powered AFOs for children with crouch gait, and highlight how subject-specific kinematics and kinetics may influence muscle demand and recruitment to inform AFO design.

## Introduction

Crouch gait, characterized by excessive knee flexion during stance, is one of the most common gait patterns among individuals with cerebral palsy (CP) [1]. Children with CP expend significantly more energy to walk than their typically-developing (TD) peers [2], which can hinder participation in activities of daily life. Increased knee flexion during crouch gait increases the muscle force required to support and propel the body [3–5], contributing to increased energy costs [6]. While many treatments aim to improve crouch gait, ankle foot orthoses (AFOs) remain one of the most common interventions. AFOs are often prescribed for children with CP to improve gait kinematics, prevent bone deformities, and potentially reduce energy costs of walking [7, 8]. However, there are many different types of AFOs and their potential to reduce energy costs of walking remains unclear.

**Fig 1:**
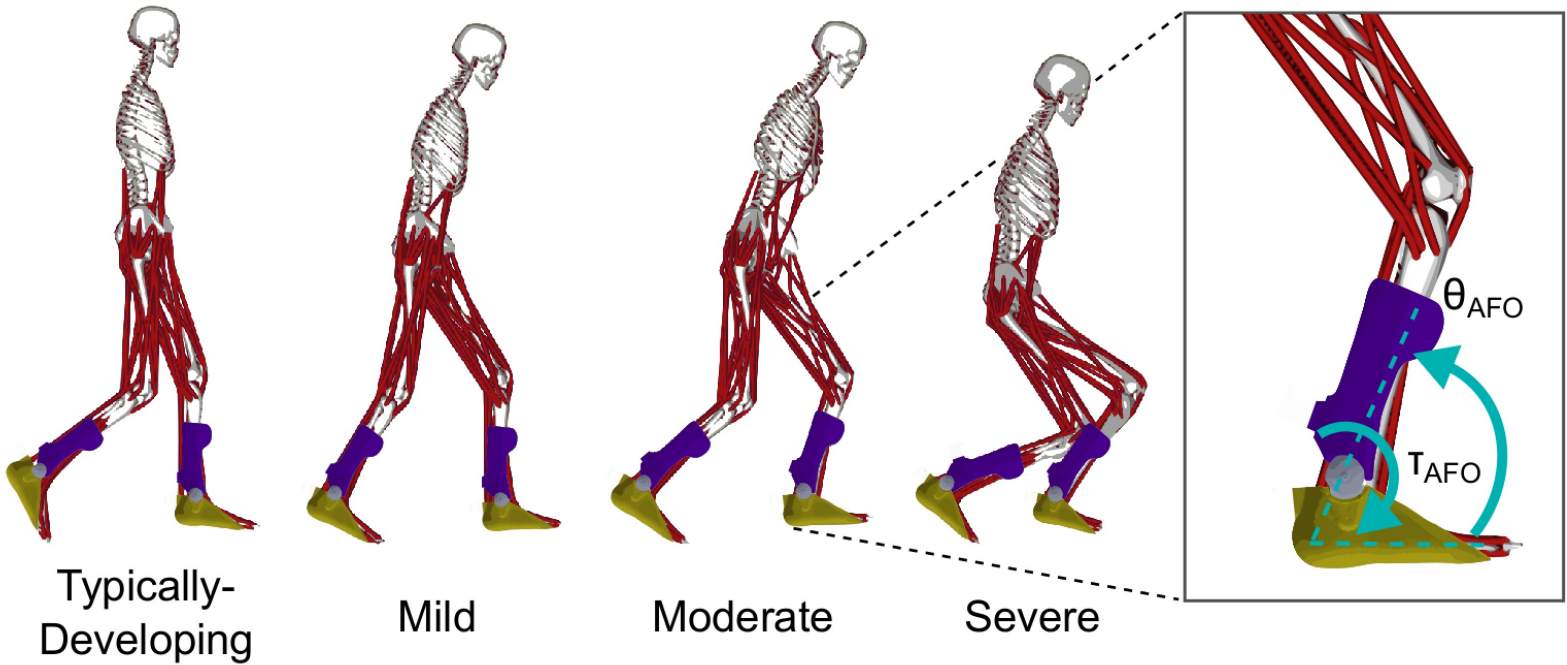
Musculoskeletal models with bilateral AFOs. The beginning of second double-limb support is shown for one typically-developing (TD) participant and one participant from each level of crouch severity. Mild (MI), moderate (MO) and severe (SE) crouch gait were defined by the minimum knee flexion angle during stance. AFO torque (*τ*_*AFO*_) was determined by AFO stiffness and AFO angle (*θ*_*AFO*_) for the passive AFOs, and by the OpenSim cost function for the powered AFOs.

Passive AFOs that resist ankle dorsiflexion are the most commonly prescribed orthoses for children with CP [9]. Solid, passive-elastic AFOs generate torque as a function of ankle kinematics and AFO properties. Two properties largely dictate the passive resistance from these AFOs: stiffness and equilibrium angle. Stiffness determines the resistance of the AFO to ankle dorsiflexion and has been shown to vary widely across passive AFO designs, with studies of CP reporting AFO stiffness ranging from 0.2–3.8 N∙m/deg [8, 10]. The AFO equilibrium angle (Fig 1) is defined as the angle between the AFO shank and footplate at which the AFO starts to generate torque. Displacement of the AFO ankle angle from the equilibrium angle produces a resistive torque proportional to the magnitude of the AFO’s angular displacement and the AFO stiffness. Orthotists often adjust these properties during AFO fabrication to customize passive AFOs for children with CP [11]. Some new AFOs even let orthotists or therapists adjust the stiffness or other properties after fabrication [12].

If designed properly, a passive AFO’s storage and release of mechanical energy can potentially reduce energetic demand during gait. Clinically-prescribed passive AFOs have been shown to reduce energy costs of walking in CP, with energy savings ranging from 6–9% compared to unassisted walking [7, 13]. Greater energetic savings have been observed using experimental AFOs. A recent study of 15 children with spastic CP tested three AFO stiffness levels and found that moderate stiffness AFOs generally provided the greatest reductions in energy costs: 11% reduction compared to unassisted walking [8]. However, predicting an individual’s response to different AFOs and identifying optimal AFO properties for each individual remains challenging [7–9, 14].

Moving beyond passive AFOs, technological advances have motivated the use of powered AFOs, which use actuators, such as electric motors, to generate torques at the ankle for assistance or augmentation [15–17]. The power generated by these AFOs has the potential to surpass achievable energy storage and return of passive AFOs during gait. Further, powered AFOs provide the opportunity to customize the timing and magnitude of the ankle torque to each individual and different activities of daily life. Unlike passive AFOs, these devices generate controllable torques that are independent of ankle kinematics, giving them the potential to further reduce energy costs of walking. In unimpaired adults, powered AFOs that assist plantarflexion have been shown to reduce energy costs by up to 11% [16, 17], as compared to 7% with passive AFOs [18]. Powered AFOs have not been tested on individuals with CP. A potential disadvantage of powered AFOs is the additional device weight and complexity. Current models often weigh two to four times that of passive AFOs in adults, and may present a larger energetic challenge for children [17, 18]. While battery packs can be mounted at the waist, much of the device mass is located distally, on the foot and shank, which can significantly increase energy costs during walking [19]. Understanding the potential energy savings of powered AFOs during pathologic gait in CP can help inform design specifications for these devices.

Experimental analyses of individuals’ responses to different AFO designs can be time-consuming and technically challenging. Measures of metabolic rate, such as oxygen consumption, are noisy and can be slow to stabilize, limiting the number of conditions that can be tested in a single session [20, 21]. Musculoskeletal simulation provides a tool to quickly perform “what-if” experiments, such as testing how different AFO designs impact muscle demand and recruitment. Recently, Uchida et al. [22] used simulation to investigate how powered lower-limb assistive devices may impact metabolic rate in adults during running. Their findings suggest that massless, powered AFOs may reduce the metabolic power required for running by up to 26%. Hegarty et al. [23] recently combined musculoskeletal simulation and probabilistic methods to evaluate the sensitivity of muscle force estimates on passive AFO mechanical properties for two children with CP. The authors demonstrated that muscle force estimates were sensitive to both AFO stiffness and equilibrium angle, further motivating the need for novel methods to predict optimal passive AFO properties for a given individual.

The goal of this research was to investigate the potential impact of powered and passive AFOs on muscle recruitment and energy costs of walking in TD children and children with crouch gait. We generated musculoskeletal simulations of children with CP and TD children, and evaluated the potential reduction in muscle force with passive and powered AFOs. We hypothesized that powered AFOs would provide greater reductions in muscle force than passive AFOs for both children with CP and TD peers. Understanding how AFOs can impact muscle demand and recruitment may motivate further investigation into the use of powered AFOs and inform AFO design for children with CP.

## Methods

### Participants

To evaluate the potential impact of AFOs on muscle recruitment during gait, we used previously-collected experimental kinematics and ground reaction force data from three TD children and nine children with diplegic CP and crouch gait (Table 1), which are available from a public data repository [24, 25]. These datasets were generated from motion analysis data originally collected at Gillette Children’s Specialty Healthcare (St. Paul, MN), and include one gait cycle of unassisted, barefoot overground walking for each participant at their self-selected speed. The participants with CP were grouped by minimum knee flexion angle (KFA) during stance, representing mild (MI; KFA 15°-30°), moderate (MO; KFA 30°-50°), and severe (SE; KFA >50°) crouch gait. These datasets were previously used in simulations to evaluate muscle contributions to mass center accelerations and tibiofemoral forces during crouch gait [5, 26].

**Table 1:** Participants (average ± one standard deviation).

		Age	Height	Mass	KFA**
	N*	(years)	(m)	(kg)	(deg)
**Typically-Developing**	3	12 ± 2	1.4 ± 0.0	38 ± 5	2 ± 1
**Mild**	3	9 ± 1	1.2 ± 0.1	24 ± 4	18 ± 3
**Moderate**	3	11 ± 2	1.4 ± 0.1	43 ± 31	34 ± 2
**Severe**	3	14 ± 2	1.6 ± 0.1	42 ± 8	64 ± 20

*N, Number of participants in each group

**KFA, Minimum knee flexion angle during stance

### Musculoskeletal modeling

To generate simulations of walking with and without AFOs, we used scaled musculoskeletal models from the original dataset. These models have 19 degrees of freedom and 92 musculotendon actuators, and were updated for compatibility with version 3.3 of OpenSim, an open-source musculoskeletal modeling program [27, 28]. We used inverse kinematics to generate joint angle trajectories by minimizing the tracking error between virtual and experimental marker trajectories. Average kinematic marker RMS error was 1.2 ± 0.2 cm and the maximum marker error was 2.8 ± 0.8 cm [29]. A residual reduction algorithm was used to improve dynamic consistency and reduce the impact of modeling and experimental errors by making small adjustments to the torso mass center position and joint angles [30]. Peak residual forces were less than 5.0% of participants’ total ground reaction forces, and peak residual moments were less than 5.9% of participants’ external moments about the center of mass. Residual forces and moments were elevated in these simulations compared to best-practice values [29] due to a number of factors. In particular, arm and torso motion can have important impacts on gait in CP [31]. Arm motion in CP has been shown to contribute to center of mass acceleration more in children with CP compared to TD children [32]. Arm motion was not modeled due to a lack of arm markers during data collection and the torso was modeled as a rigid segment. Other soft tissue artifacts can also contribute to increased residual magnitudes, but we expect arm and torso modeling assumptions to be the primary contributors to residuals in these simulations.

**Fig 2:**
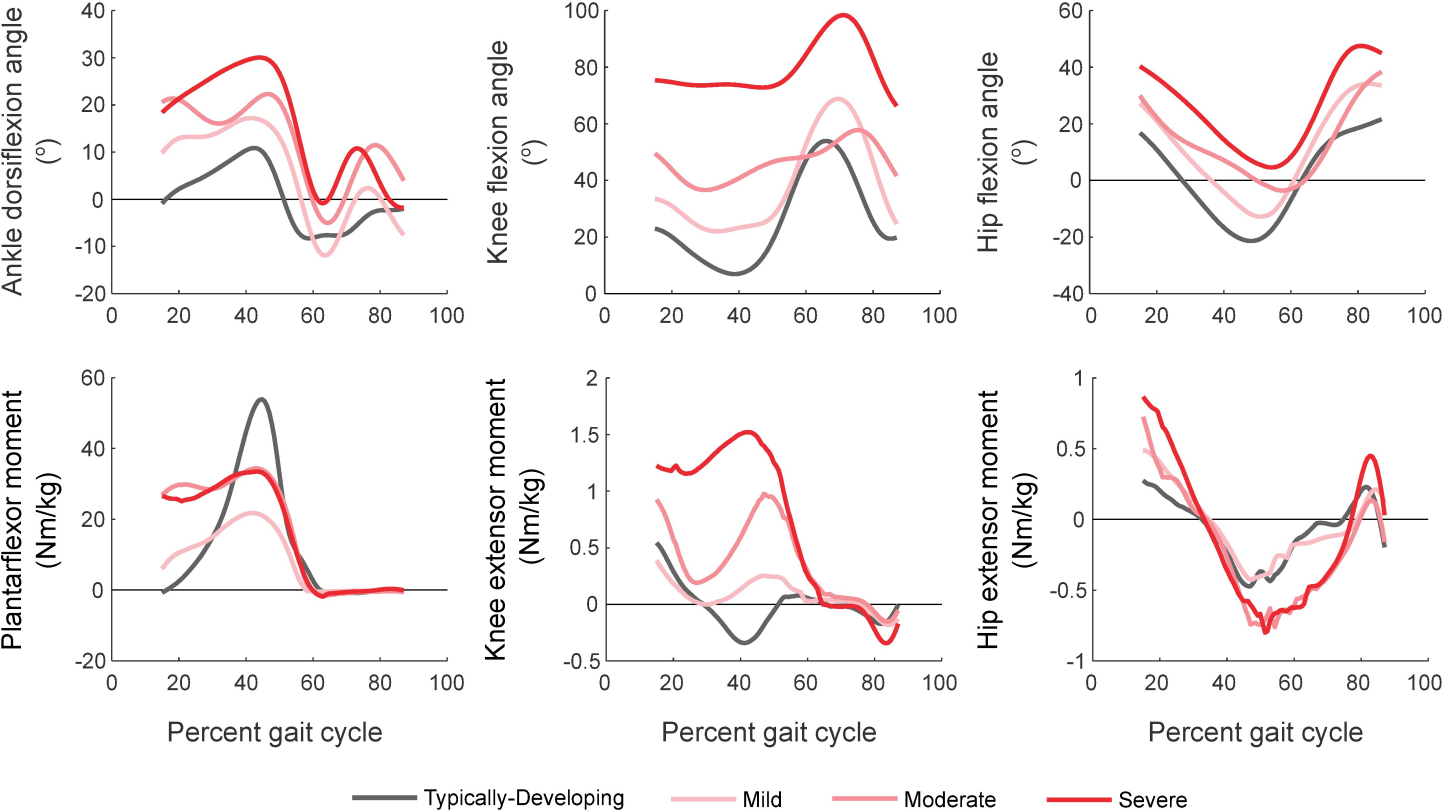
Sagittal-plane joint kinematics and internal moments. Top: Ankle, knee and hip kinematics for gait in TD children and children with crouch gait. TD children walked with less ankle dorsiflexion and knee flexion during stance than those with crouch gait. Bottom: Ankle, knee and hip moments for gait in TD children and crouch gait. TD children generated larger peak plantarflexor moments and smaller peak knee extensor moments compared to crouch gait. Knee extensor moments increased with increasing crouch severity.

We estimated muscle forces over one gait cycle using OpenSim’s static optimization algorithm. Static optimization estimates muscle forces by minimizing the sum of squared muscle activations required to generate experimental kinematics and ground reaction forces at each time point [33]. For each participant, we simulated unassisted walking and walking under four AFO conditions, using the same set of experimental kinematics and ground reaction forces (Fig 2). Although prior research has demonstrated that AFOs often produce important changes in kinematics for children with CP [7–9], changes in kinematics with existing AFOs are often small and variable between individuals [8, 9]. In this study, we aimed to understand the potential impact of AFOs on muscle demand, even if kinematics are unchanged. Thus, we assumed identical kinematics between barefoot and AFO conditions and evaluated the potential impact of AFOs on muscle force during walking.

### Quantification of muscle demand

Our primary outcome measure was the sum of muscle forces in one leg, integrated over single-limb support and second double-limb support, which we term *leg impulse*. Although this is not a direct measure of metabolic cost, the mechanical work rate of muscle is a major component of metabolic cost and is proportional to muscle force [34]. We analyzed leg impulse during single-limb support and second double-limb support for each participant, which was the range that consistently had clean ground reaction force data across all participants. Simulations showed that 85–92% of the AFOs’ impacts on muscle demand occurred during this portion of the gait cycle. This range is consistent with one experiment that reported that changes in active muscle volume during stance accounted for over 85% of the increase in metabolic rate when carrying different loads [35].

Muscle force trajectories from static optimization were processed in MATLAB (MathWorks, Inc., Natick, MA). We interpolated the entire gait cycle to 1000 data points and analyzed only single-limb support and second double-limb support. Muscle forces were normalized by each participant’s bodyweight (BW = mass∙gravity) and AFO torques were normalized by BW∙leg length [36]. For individual muscles, we defined scalar *muscle impulse* as the integral of a muscle’s force during single-limb support and second double-limb support. We computed muscle impulse for the soleus (SOL), gastrocnemius (GAS), tibialis anterior (TA), rectus femoris (RF), vasti (VAS), hamstrings (HAMS), gluteus maximus (GMAX), and iliopsoas (ILIO).

### AFO conditions

**Fig 3:**
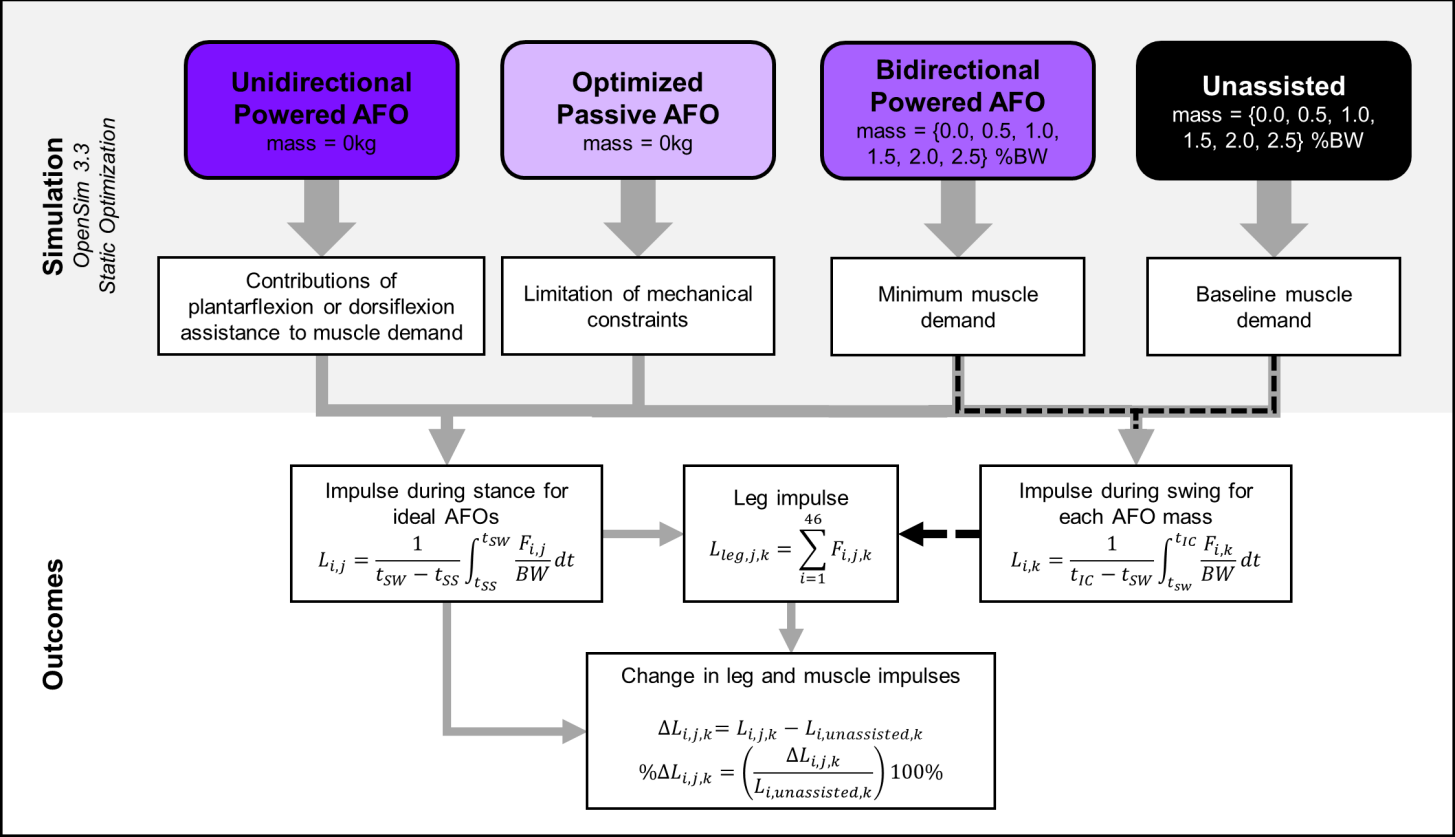
Simulation pipeline and outcome measures. Leg (*L*_*leg*,*j*,*k*_) and muscle (*L*_*i*,*k*,_ *L*_*i*,*j*_) impulses were computed for all AFO conditions, including the baseline (Unassisted) condition. Subscripts *i*, *j*, *k*, denote the muscle, AFO condition, and mass, respectively. The muscle impulses used to compare between AFO conditions were computed from the beginning of single-limb support (*t*_*SS*_) to the start of swing (*t*_*SW*_). Muscle impulses for the mass analysis (black dotted line) were computed from *t*_*SW*_ to initial contact at the start of the next gait cycle (*t*_*IC*_). Forces were normalized by bodyweight (BW). Leg impulse was computed for each participant’s leg that contained the single-limb support and second double-limb support gait phases. Change (*ΔL*_*i*,*j*,*k*_) and percent change (%*ΔL*_*i*,*j*,*k*_) in leg and muscle impulses were used to quantify changes in muscle demand between AFO conditions. *Abbreviations*: *L*_*leg*,*i*,*j*,*k*_, *Leg impulse for muscle i*, *AFO j*, *and mass k; SS*, *single-limb support phase; SW*, *swing phase; IC*, *initial contact; Δ*, *absolute change; %Δ*, *percent change*.

An overview of our simulations and analysis pipeline can be found in Fig 3. We modeled the passive AFOs as massless, sagittal-plane, constant-stiffness torsional springs at the ankle joint, resisting only dorsiflexion, similar to some AFOs used in studies of children with CP and more advanced devices used to study optimal AFO stiffness in unimpaired adults [8, 18]. The passive AFO torque profile was determined by ankle kinematics, and two AFO properties: torsional stiffness and equilibrium angle (Fig 1, right). Equilibrium angle, *θ*_*eq*_, was defined as the sagittal-plane angle between the AFO shank and footplate at which the AFO started generating torque. The passive AFO shank and footplate were fixed to the tibia and calcaneus of the model, respectively. The AFO angle, *θ*_*AFO*_, was therefore equal to the sagittal-plane ankle angle throughout the gait cycle. The plantarflexor torque generated by AFOs, *τ*_*AFO*_, was a linear function of equilibrium angle and ankle dorsiflexion angle (Eq 1). We defined the optimal combination of AFO stiffness and equilibrium angle for each participant as that which minimized leg impulse.

$$\tau_{AFO}(t)=\left\{ \begin{array}{cc} k_{AFO}∙(\theta_{AFO}(t)-\theta_{eq}(t)) & \theta_{AFO}\geq \theta_{eq}\\ 0 & \theta_{AFO}<\theta_{eq} \end{array}\right.$$ (1)

To identify optimal passive AFO properties, we simulated gait using different combinations of AFO stiffness and equilibrium angle for each participant. We performed simulations in two iterations. The first iteration simulated a grid of 400 uniformly-distributed combinations of AFO stiffness and equilibrium angle. For the children in this study, we simulated a range of AFO stiffness from 0.0 N∙m/deg to 5.0 N∙m/deg. These values were determined from studies that tested AFO stiffness values ranging from 0.7–3.8 N∙m/deg for children with CP and 0.0–7.0 N∙m/deg for unimpaired adults [8, 18]. AFO equilibrium angles ranged from minimum to maximum ankle angle during gait for each participant. We identified the combination of AFO properties from these simulations that minimized the leg impulse for each participant. We then determined the range of AFO properties around this point that resulted in less than a 5% increase in leg impulse, and simulated an additional 225 uniformly-distributed data points within this range. From these simulations, we defined the optimal AFO properties as the combination of AFO stiffness and equilibrium angle that minimized leg impulse for each participant.

We modeled the powered AFOs as additional sagittal-plane reserve actuators at the ankle joint. Unlike passive AFOs, the activation level of powered AFOs was included in and determined by the static optimization cost function. Static optimization identified the active AFO torque profile that minimized the sum of squared muscle activations during gait [37], sand thus required only a single simulation per subject for each powered AFO design. We set the optimal torque of the powered AFOs to a value of 1 MN∙m, such that the AFO torque had a negligible impact on the static optimization cost function. This optimal torque is consistent with prior work examining the impact of exoskeletons on running energetics in unimpaired adults [22], and we found that our simulation results were insensitive to further increases in the AFO’s optimal torque. We simulated two powered AFO conditions: a bidirectional AFO, which generated both plantarflexor and dorsiflexor torques, and a unidirectional AFO, which generated only assistive plantarflexor torques. We have made all musculoskeletal models, including actuators and setup files available on (https://simtk.org/projects/crouchgait).

To evaluate the impact of AFO mass, we simulated gait with AFOs with mass both with and without bidirectional actuation. We modeled each AFO as a shank piece and a footplate. The shank piece and footplate contained 66.6% and 33.3% of the AFO mass, respectively, which is consistent with experimental powered AFO designs [17]. AFO mass was increased incrementally from 0.0 to 2.5% of each participant’s bodyweight (Table 1). When AFO mass was larger than 2.5% of bodyweight, the peak and RMS residual forces from static optimization exceeded best-practice values [29], and were omitted from analysis. The foot-mounted hardware of recent powered AFOs weigh as little as 1.1 kg per foot [17]. Including the mass of a standard shoe, this would result in a total mass of approximately 2kg at each foot for children, which corresponds to 2.5–9.0% bodyweight for the children in this study. Commercial passive AFOs for children can weigh as little as 0.3 kg per foot, which, in combination with shoes, would be less than 5.0% of the mass of most children in this study.

Each AFO condition was compared to simulations of unassisted gait and AFOs were applied bilaterally for all simulations. We computed reductions in leg and muscle impulses in each AFO condition as absolute and percent change relative to the unassisted walking condition for each participant. We compared outcomes between AFO conditions by computing absolute and percent changes in leg impulse and the impulse of individual muscles. To compare the impacts of AFOs between groups, we averaged changes in leg and muscle impulses across participants in each group. The sensitivity of leg impulse to AFO mass was determined as the slope of the linear-least squares curve fit between AFO mass and leg impulse. We performed linear regression to investigate whether participant-specific parameters identified from prior research impacted changes in leg impulse. Specifically, we tested peak and average sagittal-plane lower-limb kinematics [6, 38], joint moments [38], and nondimensionalized walking speed [2]. All predictors were computed for the leg analyzed for each participant and over the same portion of the gait cycle used to compute leg impulse. We iterated through each predictor-outcome pair using a robust fitting algorithm to reduce the influence of outliers.

## Results

### Unassisted walking

**Fig 4:**
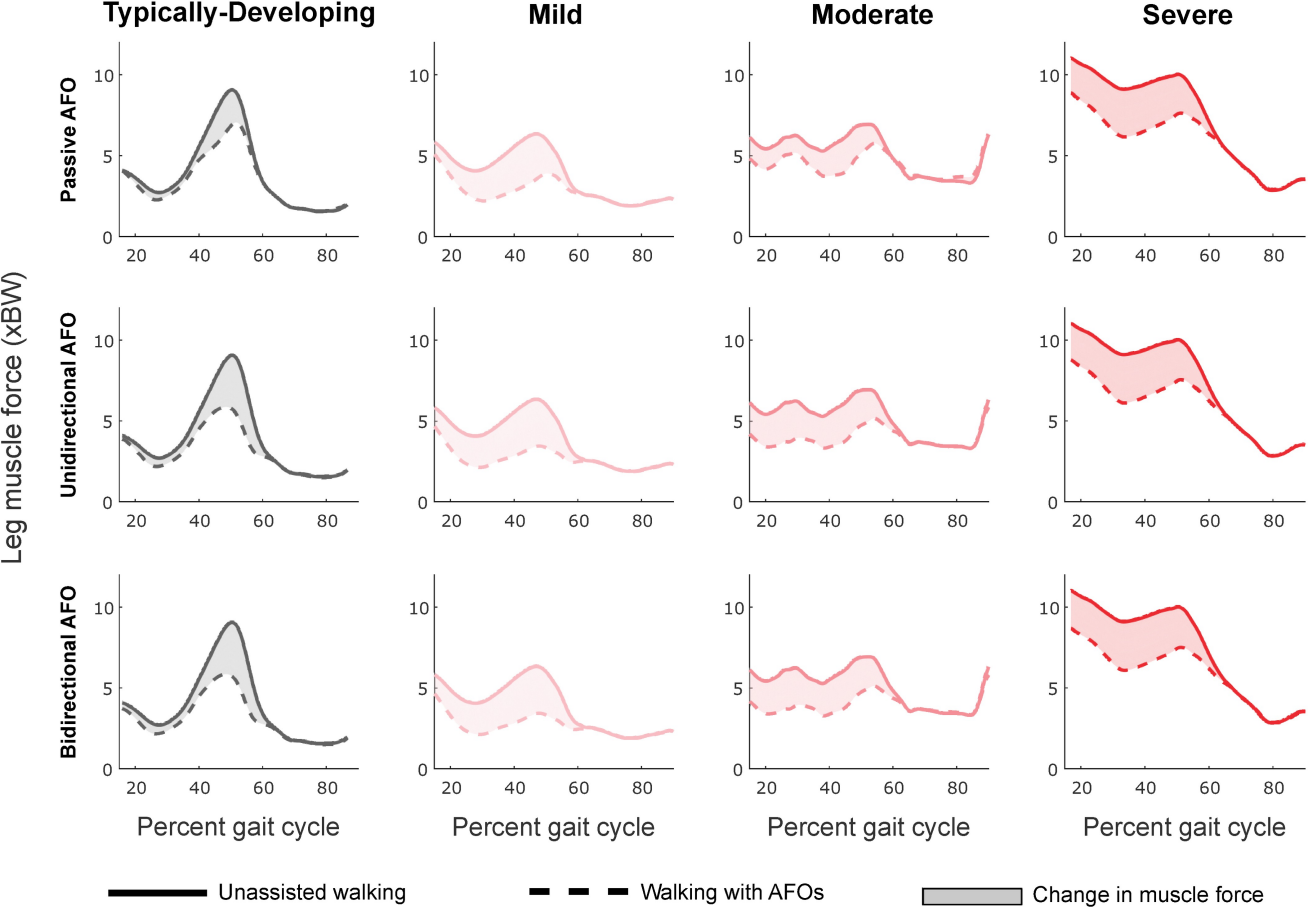
Leg muscle force with each AFO condition compared to unassisted walking. Profiles are averaged across participants in each group. Top to bottom: Optimal passive AFO, unidirectional powered AFO, and bidirectional powered AFO. The integral of these curves represents the leg impulse. Reductions in leg muscle force occurred primarily during late stance (40–60% gait cycle) for the TD group, while crouch gait groups saw leg muscle force reduced throughout single-limb support and late stance (20–60% gait cycle). Passive AFOs reduced leg muscle force less than unidirectional powered AFOs during early single-limb support for all groups, and throughout stance for the TD and moderate crouch groups. For some participants, passive and unidirectional powered AFO torque profiles were nearly identical, resulting in only small differences in leg muscle force with different AFOs. Reductions in leg muscle force were nearly identical for the unidirectional and bidirectional powered AFO conditions. Small differences in leg muscle force between these conditions occurred during swing and corresponded to changes in tibialis anterior force due to dorsiflexion assistance in the bidirectional AFO.

Crouch gait required more muscle force than gait in TD children during unassisted walking (Fig 4). Leg impulse increased with crouch severity (r^2^ = 0.80, slope = 0.06 xBW/deg, p<0.001). Impulses in the SOL (r^2^ = 0.60, slope = 0.01 xBW/deg, p<0.005), RF (r^2^ = 0.44, slope = 0.004 xBW/deg, p<0.001), GMAX (r^2^ = 0.84, slope = 0.003 xBW/deg, p<0.001) and VAS (r^2^ = 0.92, slope = 0.04 xBW/deg, p<0.001) increased with crouch severity, while GAS impulse decreased with increasing crouch (r^2^ = 0.47, slope = -0.004 xBW/deg, p<0.02). HAMS impulse was uncorrelated with crouch severity for these participants (r^2^<0.06).

### Passive AFOs

**Fig 5:**
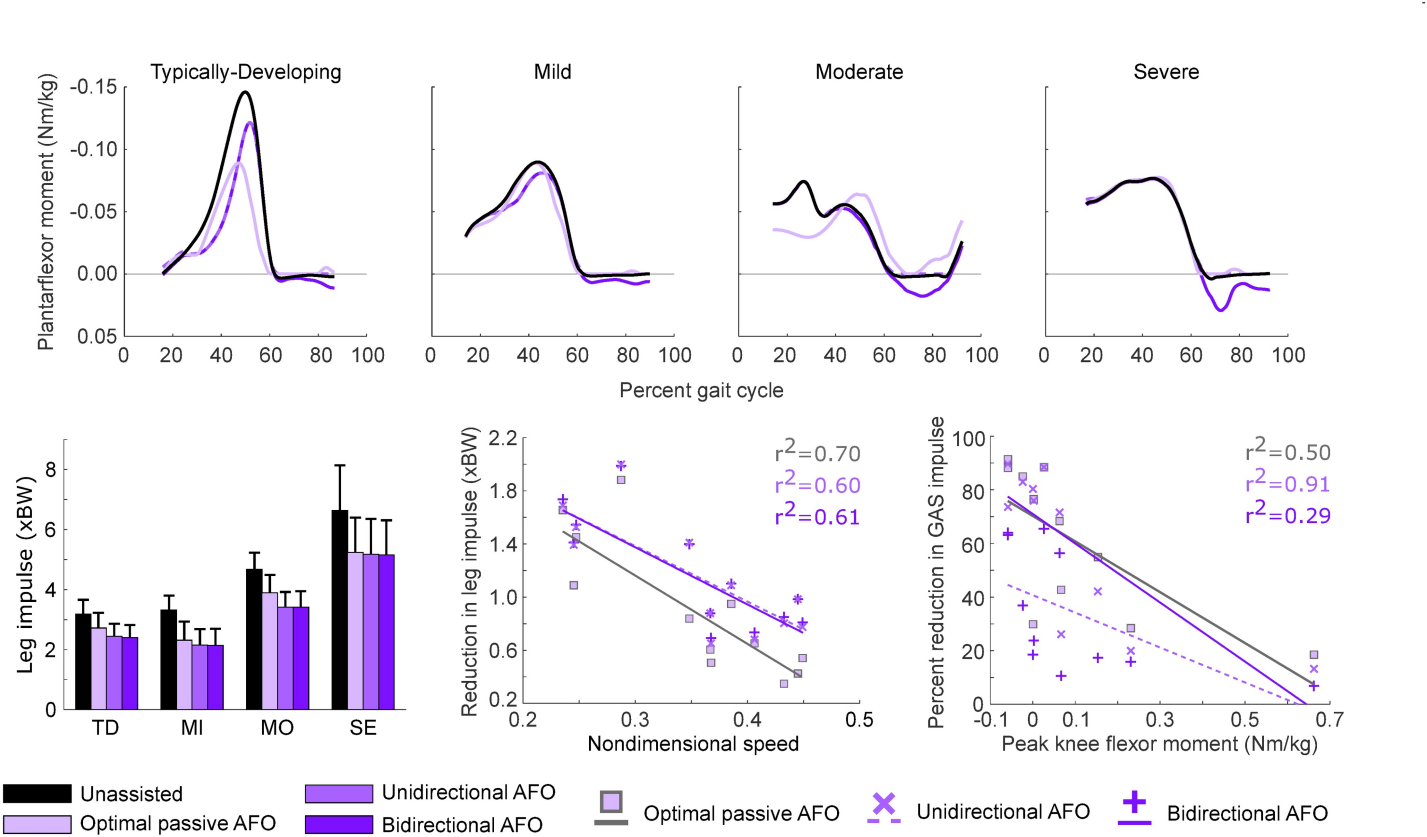
AFO torque profiles, leg impulse for each AFO condition and predictors of reductions in leg and muscle impulses. Top: Net ankle moments determined by inverse dynamics and AFO torque profiles for gait in TD children and children with crouch gait. A positive moment corresponds to a plantarflexor torque. Bottom, left: Leg impulse magnitude increased with crouch severity. Bottom, center: Reduction in leg impulse was strongly correlated with nondimensional speed for all AFO conditions. Bottom, right: GAS impulse was most strongly correlated with peak knee flexor moment. GAS activity in one subject with severe crouch was estimated to be near-zero during stance, and was omitted from this figure. *Abbreviations*: *TD*, *typically-developing; MI*, *mild crouch; MO moderate crouch; SE; severe crouch; GAS*, *gastrocnemius muscle group*.

Passive AFOs that resisted dorsiflexion reduced muscle demand during gait for all participants, primarily during terminal stance for TD participants and throughout stance for crouch gait (Fig 4). Compared to unassisted walking, leg impulse was reduced 15% in the TD group with passive AFOs, and 31, 17 and 21% in the mild, moderate and severe crouch groups, respectively (Fig 5, Table 2). Percent reduction in leg impulse was not correlated with crouch severity (r^2^<0.01), nor was it strongly correlated with any of the predictors selected from prior studies. However, the absolute reduction in leg impulse increased moderately with increasing crouch severity (r^2^ = 0.40, slope = 0.01 xBW/deg, p<0.03), with gait in TD children having the smallest impulse reduction (0.5 xBW). Nondimensional speed (r^2^ = 0.70, slope = -5.20 xBW, p<0.001) and average ankle angle (r^2^ = 0.70, slope = 0.03 xBW/deg, p<0.001) were the strongest correlates with absolute reduction in leg impulse. Passive AFO torque profiles did not resemble net ankle moments for TD and moderate crouch gait (Fig 5, top).

**Table 2:** Reduction in leg impulse versus unassisted gait, showing average absolute (xBW, ± SD) and percent change (± SD) in leg impulse.

	Typically-Developing	Mild	Moderate	Severe
**Passive AFO**	0.5 ± 0.1	1.0 ± 0.4	0.8 ± 0.3	1.4 ± 0.7
	15 ± 5%	31 ± 13%	17 ± 7.8%	21 ± 9%
**Powered AFO, Unidirectional**	0.7 ± 0.1	1.2 ± 0.3	1.3 ± 0.2	1.5 ± 0.7
	12 ± 2%	36 ± 11%	27 ± 5%	22 ± 9%
**Powered AFO, Bidirectional**	0.8 ± 0.1	1.2 ± 0.3	1.3 ± 0.2	1.5 ± 0.7
	25 ± 2%	36 ± 11%	27 ± 5%	22 ± 9%

**Fig 6:**
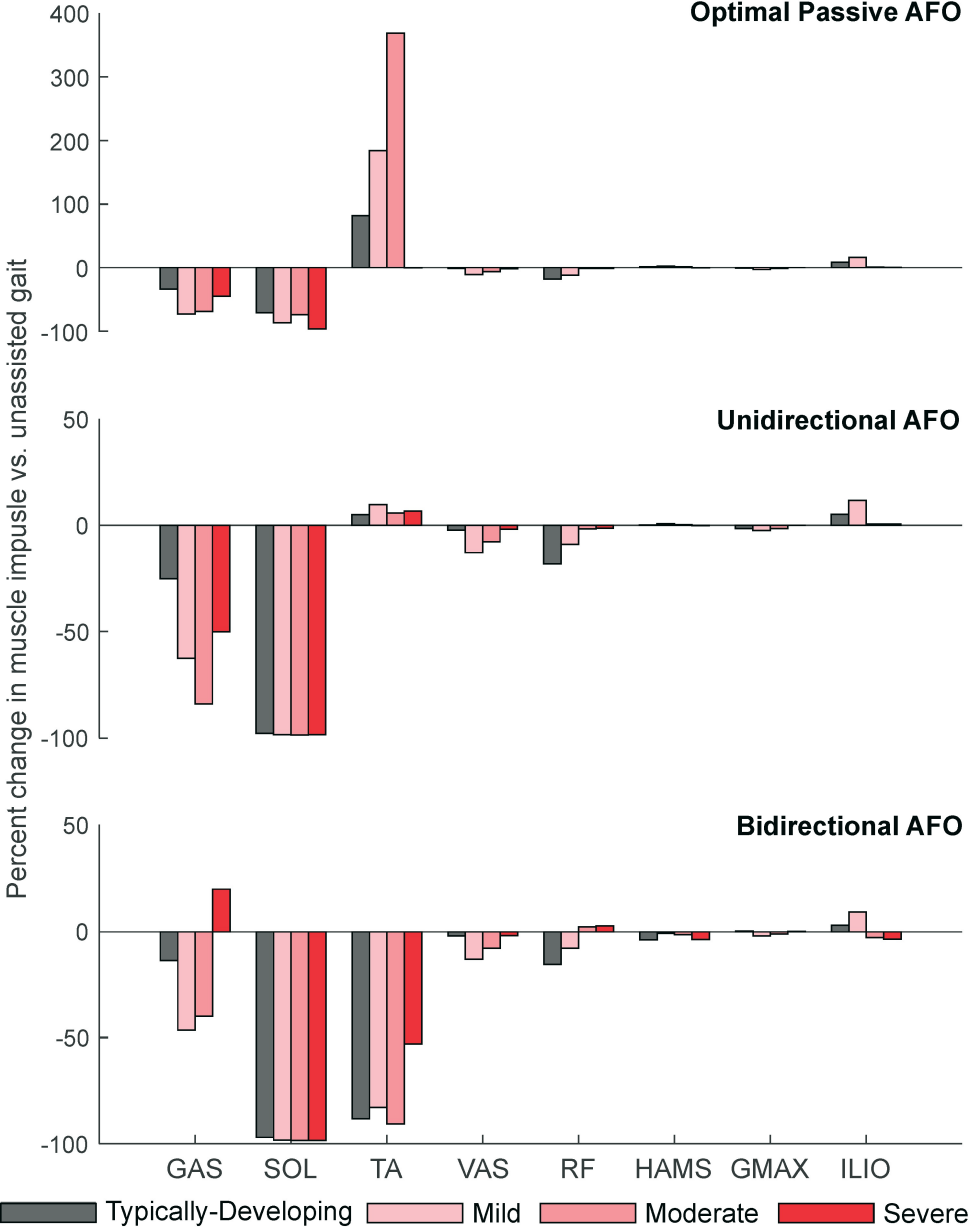
Percent change in impulse of individual muscles across AFO conditions. The GAS and SOL were most impacted by all AFO conditions, with relatively small changes occurring in other muscles. Top: The TA impulse increased with passive AFOs to overcome the AFO’s dorsiflexion resistance and maintain ankle kinematics. The large percent increase in TA impulse for the optimized passive AFO corresponds to only a small absolute increase in TA impulse compared to unassisted gait. Middle: The unidirectional powered AFO had similar reductions in muscle impulses as the bidirectional AFO, except for the GAS. Bottom: Only the bidirectional powered AFO reduced TA impulse, but this corresponded to a smaller percent reduction in GAS impulse. Impulses in muscles spanning the knee and hip changed by less than 20%, with the VAS having the largest reductions in muscle demand within these groups. A*bbreviations*: *GAS*, *gastrocnemius; SOL*, *soleus; TA*, *tibialis anterior; VAS*, *vasti; RF*, *rectus femoris; HAMS*, *biarticular hamstrings; GMAX*, *gluteus maximus; ILIO*, *iliopsoas*.

For individual muscles, only the ankle plantarflexors had a reduction in muscle impulse of more than 20% with passive AFOs compared to unassisted gait (Fig 6). Passive AFOs reduced GAS impulse by 34–69% and SOL impulse by 71–97%. Percent reduction in GAS impulse with passive AFOs decreased with increasing peak knee flexor moment (r^2^ = 0.50, slope = -98.0 xBW/(N∙m/kg), p<0.02). TA impulse increased with passive AFOs for the TD, mild and moderate crouch groups (82–369%), but this change corresponded to only a 0.03–0.14 xBW absolute increase in impulse. Reductions in VAS (2–11%) and RF (1–18%) impulses largest for muscles not spanning the ankle, and were variable between groups with passive AFOs.

The average AFO stiffness that produced the greatest reduction in leg impulse was 4.6 ± 1.4 N∙m/deg for gait in TD children and 2.3 ± 1.4 N∙m/deg for crouch gait. Normalizing AFO stiffness by participant mass resulted in different optimal stiffness values in each CP group: 0.12 ± 0.04 N∙m/(kg∙deg) for gait in TD children and 0.09 ± 0.06, 0.05 ± 0.02, and 0.05 ± 0.02 N∙m/(kg∙deg) for the mild, moderate, and severe crouch gait, respectively. The normalized passive AFO stiffness correlated most strongly with peak ankle plantarflexor moment (r^2^ = 0.75, slope = -0.11 deg^-1^, p<0.001). The AFO equilibrium angle that resulted in the largest reduction in leg impulse was largest for severe crouch (12.8 ± 7.3 deg) and smallest for gait in TD children (3.5 ± 5.6 deg). This angle increased (*i*.*e*., a more dorsiflexed equilibrium angle) as peak plantarflexion angle decreased (r^2^ = 0.88, slope = -0.4, p<0.001).

### Powered AFOs

Unidirectional powered AFOs reduced leg impulse for all subjects, with an average reduction of 23 ± 2% and 28 ± 10% compared to unassisted gait for the TD and crouch groups, respectively. This corresponds to a 10 ± 4% and 7 ± 5% greater reduction than passive AFOs for the TD and crouch groups, respectively. The largest reductions in leg impulse with the unidirectional powered AFO occurred during single-limb support for all groups, and throughout stance for the TD and moderate crouch groups (Fig 4). Large reductions in leg impulse with the unidirectional powered AFO represent the benefit of powered AFOs over passive AFOs of comparable design. However, the difference in leg impulse reductions between AFO designs was variable, with participants experiencing a 1% to 13% difference between powered and passive AFOs. Leg impulse reductions were smallest for participants whose passive AFO torque profiles most closely matched their unidirectional powered AFO torque profiles. Minimum KFA during stance was moderately correlated with the absolute reduction in leg impulse (r^2^ = 0.41, slope = 0.01 xBW/deg, p<0.03), but was not correlated to percent reduction in leg impulse (r^2^<0.07). Similar to passive AFOs, nondimensional speed (r^2^ = 0.60, slope = -4.21 xBW, p<0.004) was most strongly correlated with the absolute reduction in leg impulse.

Walking with bidirectional powered AFOs reduced leg impulse similar to the unidirectional powered AFOs (Table 2, Fig 5). During gait in TD children, the average reduction in leg impulse was 25%, while leg impulse was reduced 36, 27 and 22% for mild, moderate and severe crouch groups, respectively, compared to unassisted walking (Table 2, Fig 5). Bidirectional powered AFOs assisted both ankle plantarflexion and dorsiflexion as much as needed, and thus returned the maximum achievable reductions in leg impulse from our simulations. However, dorsiflexion assistance corresponded to only a 1% greater reduction in leg impulse compared to the unidirectional AFOs. Regression results were nearly identical to the unidirectional AFO condition: Percent reductions in muscle demand were not correlated with crouch severity (r^2^<0.05), or any other pre-selected predictor. Participants with greater crouch severity had larger reductions in leg impulse: 0.8, 1.2, 1.3 and 1.5 xBW for gait in TD children and mild, moderate and severe crouch gait, respectively. Minimum KFA during stance was moderately correlated to these reductions (r^2^ = 0.43, slope = 0.01 xBW/deg, p<0.03). Similar to the passive AFO, average ankle angle (r^2^ = 0.70, slope = 0.03 xBW/deg, p<0.001) and nondimensional speed (r^2^ = 0.61, slope = -4.20 xBW, p<0.003) were most strongly correlated to absolute reduction in leg impulse. Both bidirectional and unidirectional AFO torque profiles closely resembled net ankle moments for crouch gait and more closely resembled net ankle moments compared to passive AFOs for gait in TD children (Fig 5, top).

Similar to passive AFOs, the powered AFOs primarily reduced muscle demand in the ankle plantarflexors, with reductions in muscle impulse of more than 20% in only the GAS and SOL for both powered AFOs, as well as in the TA for the bidirectional AFO (Fig 6). Unlike the passive AFO condition, SOL impulse reduction was similar for all crouch groups (97–98%) for both powered AFO conditions. The percent reduction in GAS impulse decreased with increasing peak knee flexor moment for the unidirectional AFOs (r^2^ = 0.91, slope = -225.0%/(N∙m/kg), p<0.001) and bidirectional AFOs (r^2^ = 0.29, slope = -65.3%/(N∙m/kg), p<0.1). Percent reductions in GAS impulse ranged from 25–84% for the unidirectional AFO condition, and 13–46% for the bidirectional AFO condition. The smaller reduction in GAS impulse with the bidirectional AFO compared to unidirectional AFOs was due to the bidirectional AFOs’ dorsiflexion assistance: By generating a dorsiflexor torque during swing, the bidirectional AFOs enabled the GAS to contribute to knee flexion moments more than during unassisted walking, reducing demand on the HAMS (Fig 6). Consequently, bidirectional assistance resulted in small reductions in both the TA and HAMS impulses in these simulations. Minimum KFA was correlated with the reduction in GAS impulse only for the unidirectional AFOs (r^2^ = 0.57, slope = -1.07 xBW/deg, p<0.01). TA impulse was reduced 53–91%, only when walking with bidirectional powered AFOs. Powered AFOs’ impacts on muscles spanning the knee and hip were similar to those of passive AFOs, with the VAS (2–13%) and RF (1–18%) muscles showing the largest reductions in impulse compared to unassisted gait. Although the change was small (<5.0%), the bidirectional AFO was the only device to reduce HAMS recruitment compared to unassisted gait.

Leg impulse increased with increasing AFO mass in all participants, but sensitivity to AFO mass decreased with increasing crouch severity. Leg impulse increased at a rate of 0.14, 0.18, 0.15, 0.10 xBW/kg_AFO_ for the gait in TD children, and mild, moderate and severe crouch groups, respectively (Table 3). Powered AFO actuation increased sensitivity to AFO mass by less than 0.03 xBW/kg_AFO_ compared to the unactuated AFO. Increases in muscle impulse due to AFO mass occurred primarily in the knee flexors and hip flexors during swing, but were not correlated with minimum KFA during stance. Hip flexor force increased in early swing, while knee flexor force increased most during mid and late swing.

**Table 3:** Sensitivity of leg impulse to AFO mass (xBW/kg_AFO_).

	Mass sensitivity (xBW/kg_AFO_)*	
	Unactuated	Bidirectional
**Typically-Developing**	0.14 ± 0.06	0.15 ± 0.06
**Mild**	0.18 ± 0.05	0.21 ± 0.05
**Moderate**	0.15 ± 0.10	0.17 ± 0.11
**Severe**	0.10 ± 0.08	0.10 ± 0.09

*Sensitivity is defined as the slope of the leg impulse vs. AFO mass curve.

## Discussion

We simulated the effects of passive and powered AFOs on muscle demand and recruitment during walking in TD children and children with CP and crouch gait. We hypothesized that powered AFOs would reduce leg impulse more than passive AFOs. The simulation results supported this hypothesis, with leg impulse being reduced 1–15% more with powered AFOs than passive AFOs, supporting the potential use of powered AFOs as assistive devices for CP. Unlike passive AFOs, powered AFO torque profiles are independent of ankle kinematics which increases the ability to customize torque profiles to an individual’s gait pattern. We also found, as anticipated, that all AFO designs primarily impacted the ankle plantarflexor muscles; however, reductions in muscle impulse were only moderately correlated with crouch severity, emphasizing the diverse factors that influence an AFO’s impact on muscle demand, even among children with similar gait patterns.

As idealized models of passive and powered AFOs, the results of this study provide an estimate of the potential of AFOs to reduce muscle demand during gait for children with CP independent of changes in kinematics. Prior studies with passive AFOs reported reductions in metabolic cost ranging from 6–10% [7, 8], though no prior studies have investigated the impact of powered AFOs on muscle demand in CP. In this study, we observed 10–41% reductions in leg impulse with AFOs, which is one of the primary contributors to metabolic cost of walking. However, there are other factors such as cardiovascular health or selective motor control which may influence reductions in metabolic cost beyond reductions in muscle demand. Our results suggest that appropriate tuning of AFO mechanical properties to an individual may optimize reductions in muscle demand for children with CP. Further, the results from this study highlight the importance of optimizing AFOs across multiple parameters to minimize muscle demand during walking. Passive AFO optimization protocols have been proposed, but typically select between only a few different AFO stiffness values or designs [39, 40], possibly limiting the “optimality” of selected AFO properties.

Identifying the optimal AFO properties for a given individual remains an open challenge, though musculoskeletal simulation may be used to inform subject-specific device design. Testing over many potential AFO tuning parameters is time and resource intensive, making simulation-based predictions of AFOs attractive. We found that absolute reductions in leg impulse with all AFO designs correlated most strongly with nondimensional speed, and that percent reductions in GAS impulse correlated most strongly with peak knee flexor moments. This highlights the importance of activity-level (walking speed) and mechanical-level (kinematics, kinetics) measurements in predicting the impacts of AFOs on gait in CP [41]. The complex interaction between mechanical and activity-level aspects of gait suggests that a multivariate approach to predicting AFO impacts on muscle demand and recruitment is necessary. A post-hoc multiple linear regression analysis found that a linear combination of lower-limb kinematics and joint moments was strongly correlated with both absolute and percent reductions in muscle demand. This is consistent with one study that found that a similar linear combination of mechanical-level measures explained 89% of the variance in net metabolic cost during uphill walking in unimpaired adults [38]. While our regression analysis suggested that nondimensional speed may be a useful way to quantify the potential impact of AFOs on muscle demand if kinematic or kinetic data are unavailable, new methods are needed to evaluate the potential benefit of AFO designs. The simulation paradigm presented in this research may be used to help identify a near-optimal set of mechanical AFO properties for an individual and potentially inform biofeedback or other training regimes to optimize muscle recruitment while walking with a new AFO.

There were similarities in the optimal AFO properties predicted in our study and prior experimental results. The optimal passive AFO stiffness values predicted for the TD children were larger than a prior study that identified the passive AFO stiffness that most reduced metabolic costs in unimpaired adults (4.6 N∙m/deg vs 3.1 N∙m/deg [18]). Similarly, for crouch gait, our optimal stiffness of 2.3 N∙m/deg was slightly larger than a reported optimal of 1.6 N∙m/deg for passive AFOs that minimized energy costs of walking in CP [8], though this study evaluated three AFO stiffness conditions with a maximum stiffness of 3.8 N∙m/deg. These differences may be due in part to the instantaneous “adaptation” to AFOs that occurs during simulation, compared to slow and complex motor adaptation in human gait. Further, simulated AFO stiffness has been shown to alter the strain of elastic elements in the ankle plantarflexors, which leads to changes in metabolic power in these muscles during gait [42]. Consistent with our findings, this suggests that the comparatively simple musculotendon model used in static optimization may over-predict optimal AFO stiffness values. Passive AFO equilibrium angle is less studied than AFO stiffness [43], but correlated well with peak ankle plantarflexion angle. Thus, basing passive AFO equilibrium angle on ankle kinematics may generate a good initial guess prior to tuning AFOs to minimize muscular demand during gait. The range of optimal AFO properties found in this study emphasizes the importance of appropriate AFO actuator design and mechanical properties based on an individual’s gait pattern.

Powered AFOs outperformed passive AFOs in this study and may be more effectively tuned to a specific individual. However, their use may not be justifiable if powered AFOs are too heavy. Consider that our reported sensitivity of leg impulse to AFO mass (~0.1 xBW/kg_AFO_) was similar to the contribution of powered AFO dorsiflexion assistance to reductions in leg impulse (0.1–0.2 xBW). This implies that the additional hardware required for powered dorsiflexion assistance would have to weigh less than 1kg to reduce leg impulses. Moreover, some participants received only a small (<3%) benefit of powered AFO assistance over passive assistance. Since powered AFOs can weigh four times as much as passive AFOs, the additional weight of powered actuation may not be justifiable if energetic savings is a primary objective [17, 18]. The small linear increase in muscle demand due to AFO mass applied to the foot and shank was qualitatively consistent with experimental studies of unimpaired walking that found that metabolic rate increased linearly with mass added to the foot and shank [19]. This suggests that results from small AFO masses may be extrapolated to masses larger than 2.5% of participants’ bodyweights. For example, our results suggest that the weight of powered AFOs [17] may increase leg impulse by 10% for a child with mild CP, which could eliminate the potential advantage of powered AFOs over passive AFOs in reducing muscle demand during walking. However, the sensitivity to AFO mass decreased as crouch severity increased, which may be explained by a reduced mass moment of inertia of the AFO mass about the hip and knee in the sagittal plane [44]. Thus, individuals with more severe crouch gait, whose limbs may have less rotational inertia with about the hip, may be less sensitive to mass added to the foot and shank. These results collectively suggest that AFO actuator design, mass, and mass distribution should all be considered and informed by an individual’s gait analysis.

There are also other important factors that may limit achievable reductions in metabolic costs with AFOs for children with CP. In particular, children with CP have been shown to have less complex motor control strategies than their TD peers, which may limit their ability to adapt muscle recruitment to AFOs [45, 46]. If muscle coordination is restricted to synergistic motor patterns, a child may not be able to modulate muscle activity and take advantage of the potential energy savings. Prior studies with varying AFO properties for children with CP have provided short training periods, which may also limit resulting changes in muscle activity and reductions in metabolic cost. The results of this study support the potential of passive and powered AFOs to reduce energy costs in CP, but highlight the need to evaluate changes in neuromuscular control and train appropriate changes in muscle recruitment with assistive devices.

AFOs primarily impacted ankle plantarflexor activity and the results of this study demonstrate the complex and important role these muscles play during gait. Prior experimental studies of unimpaired adults have demonstrated a reduction in ankle plantarflexor activity with both passive and powered AFOs [15, 18, 47]. However, prior experimental studies have not investigated changes in plantarflexor muscle activity with AFOs in children with CP [7, 8, 23]. We found that reductions in GAS recruitment were not only important for ankle dynamics, but also correlated with peak knee flexor moments, which decreased with crouch severity [48]. This highlights the potential of AFOs to indirectly influence knee flexor moments. Reduced GAS recruitment with simulated AFOs also reduced the GAS’s contribution to knee flexor moments during stance, which in turn reduced the demand of the knee extensor muscles. This effect was reflected in reductions in VAS and RF impulses with all AFOs. Since the knee extensors are active during most of stance in crouch gait [5], a smaller knee flexor moment contribution of the GAS may also enable the knee extensor muscles to further extend the knee, possibly enabling a less crouched posture. We must acknowledge, however, that crouch gait can arise from myriad factors. Simply reducing knee extensor demand may not reduce crouch [49, 50]. For example, if short hamstrings contribute to crouch, reduced demand on the knee extensors with AFOs may not improve crouch [51]. Conversely, if coactivation or spasticity influences crouch, altered muscle demand with AFOs may enable the quadriceps to more effectively extend the knee. These results may also inform future studies of myoelectric control of powered AFOs for children with CP. Myoelectric feedback control of powered AFOs typically uses SOL activation signals for control during unimpaired gait [15, 52]. Using GAS activation signals for feedback control of powered AFOs may enable children with crouch gait to better influence both knee and ankle dynamics.

The results of this study should be taken in the context of the study’s limitations. Our inverse simulations were constrained by one set of experimental kinematics and kinetics for each individual to evaluate potential reductions in muscle demand independent of changes in gait pattern. In contrast, clinically-prescribed passive AFOs are often designed to alter gait kinematics, and powered AFOs have been shown to alter lower-limb joint moments compared to unassisted walking [15, 47]. For example, allowing powered AFOs to reduce extensor moments at the knee may enable greater simulated reductions in knee extensor impulse compared to unassisted gait. Predicting changes in gait after an intervention remains a grand challenge of biomechanics which may further enhance our ability to identify optimal, customized orthoses. Maintaining constant kinematics and kinetics across AFO conditions provides a reasonable method to evaluate potential energy savings of a wide range AFO designs, prior to experimentation [9, 22]. Also, we used OpenSim’s static optimization algorithm to estimate muscle activity, which does not model excitation-activation or tendon dynamics, both of which may influence muscle force during dynamic tasks [42, 53]. Conversely, OpenSim’s computed muscle control algorithm (CMC) [30] includes tendon dynamics, but over-predicts muscle forces [22, 23]. We compared these algorithms and found that CMC predicted greater overall muscle activity, and our outcome measures and conclusions were insensitive to algorithm choice. Finally, bone deformities, contracture, and spasticity are common in CP and were not captured in our models. This dataset was originally selected to include participants with minimal bone deformities [54], but minimal information regarding muscle physiology and spasticity were available for these participants. Incorporating individual changes in muscle properties, such as contracture or weakness, may further improve the ability of these methods to predict optimal AFO design for a given individual. Future simulation-based orthosis optimizations may also want to consider maximum allowable reductions in muscle activity to help prevent atrophy or exacerbate muscle weakness. Even with more accurate subject-specific modeling and simulation methods, researchers should acknowledge the importance of patient feedback in the AFO prescription process. Device users provide important feedback that is not captured by musculoskeletal models, such as comfort or interactions of soft-tissue with the AFO. The simulation pipeline presented in this work can be used to complement clinician expertise and help customize AFO design for rehabilitation and performance goals.

## Conclusion

Optimizing the design of powered or passive AFOs has the potential to reduce muscle demand and improve metabolic efficiency for children with CP, even without changes in an individual’s gait pattern. These changes are clinically important because children with CP have inefficient gait patterns compared to TD peers and optimizing AFOs to reduce energy costs may reduce fatigue and increase participation in daily life. Musculoskeletal simulation provides a platform to evaluate and test AFO designs and inform training by predicting optimal patterns of muscle recruitment. Although crouch gait represents one of the most common gait pathologies in CP [1], many other common gait pathologies exist that could benefit from similar analyses. Further understanding of the role of concomitant impairments such as muscle weakness, spasticity, or contracture represent important areas for future investigation. To encourage expansion of musculoskeletal simulation to assistive device applications, we have made our simulations available for others to use and build upon (https://simtk.org/projects/crouchgait). Adaptation to and optimization of AFOs remain challenging topics [9, 52], and future work comparing predictions with experimental tests will further enhance these methods. This study informs future clinical design and prescription of AFOs for children with CP and motivates further investigation into powered AFOs as assistive devices for children with CP.
